# Nervous System-Driven Osseointegration

**DOI:** 10.3390/ijms23168893

**Published:** 2022-08-10

**Authors:** Ruoyue Sun, Long Bai, Yaru Yang, Yanshu Ding, Jingwen Zhuang, Jingyuan Cui

**Affiliations:** 1 Key Laboratory for Ultrafine Materials of Ministry of Education, College of Materials Science and Engineering, East China University of Science and Technology, Shanghai 200237, China; 2 Institute of Translational Medicine, Shanghai University, Shanghai 200444, China; 3 Frontiers Science Center for Materiobiology and Dynamic Chemistry, East China University of Science and Technology, Shanghai 200237, China; 4 College of Materials and Textile Engineering, Jiaxing University, Jiaxing 314001, China

**Keywords:** nervous system, neurotrophin, neuropeptide, nerve cell, osseointegration

## Abstract

Implants are essential therapeutic tools for treating bone fractures and joint replacements. Despite the in-depth study of osseointegration for more than fifty years, poor osseointegration caused by aseptic loosening remains one of the leading causes of late implant failures. Osseointegration is a highly sophisticated and spatiotemporal process in vivo involving the immune response, angiogenesis, and osteogenesis. It has been unraveled that the nervous system plays a pivotal role in skeletal health via manipulating neurotrophins, neuropeptides, and nerve cells. Herein, the research related to nervous system-driven osseointegration was systematically analyzed and reviewed, aiming to demonstrate the prominent role of neuromodulation in osseointegration. Additionally, it is indicated that the implant design considering the role of neuromodulation might be a promising way to prevent aseptic loosening.

## 1. Introduction

Bone implants are widely used in orthopedics and dentistry to reduce pain and rehabilitate bone function [1]. However, implant failure that needs further revision remains a significant clinical challenge, resulting in higher complications and mortality rates. Aseptic loosening is the most common factor for revision surgeries, accounting for one-third of such surgeries [2]. For a long-term and reliable fixation of implants, stable osseointegration is an efficient solution to prevent aseptic loosening, that is, a direct structural and functional connection between the host bone and the surface of an implant [3]. Accordingly, numerous strategies, such as surface modification, bioactive ion loading, and mechanical optimization, have been developed to accelerate osseointegration to achieve a rapid and firm fixation with the bone tissue [2]. However, inconsistent results among the in vitro and in vivo studies have indicated that the underlying mechanism of osseointegration is still unclear [4]. It has been demonstrated that the process is initiated by blood clot formation, is then regulated by osteoimmunomodulation, and subsequently by angiogenesis and osteogenesis [5,6]. It is a continuous process involving various biological reactions, numerous cells, and mediators [7].

Nerve tissue, the main component of the nervous system, is composed of neurons (nerve cells) and glia. The neuron has the function of receiving stimulation and conducting excitation. It is also the basic functional unit of nerve activity. Glia play a supporting, protective, and nutritional role in nerve tissue. After being stimulated, neurons can produce excitation and conduct excitation along nerve fibers, and thus affect the innervated tissues. Studies have shown that neurotrophins, neuropeptides, and nerve cells affect skin wound-healing [8], pregnancy and brain development [9], inflammation, and allergies [10]. It is worth noting that the nervous system is widely distributed in bone tissue. Nerve fibers are scattered throughout the membrane, cortical bone, cancellous bone, and the marrow [11]. Of note, nerve fiber terminals are inserted around the blood vessels in the bone trabecula and bone marrow, according to a study on the distribution of nerve fibers in bone tissue [11], which indicates that the nervous system plays an important role in bone remodeling.

Once implanted, the bone tissue’s nerve system is injured. A scarce amount of research has investigated the function of the nerve fibers in bones due to their scarcity in numbers compared to other tissues. It is also challenging to locate nerves in the bone due to technical limitations. However, it has been recognized that the nervous system plays a pivotal role in bone development and fracture healing [12]. Moreover, an increasing number of studies have provided insights into the link between the nervous system and osseointegration and continue to develop the concept of implant-mediated sensory-motor control [13], which has substantial therapeutic implications but has generally been overlooked for decades. Accordingly, the present review aims to summarize the compiled knowledge about the pivotal role of the nervous system in different stages throughout the process of osseointegration, which will reference the systematic and rational design of the implants and how they achieve rapid and fulfilled osseointegration.

## 2. Osseointegration

### 2.1. The Concept of Osseointegration

**Figure 1:**
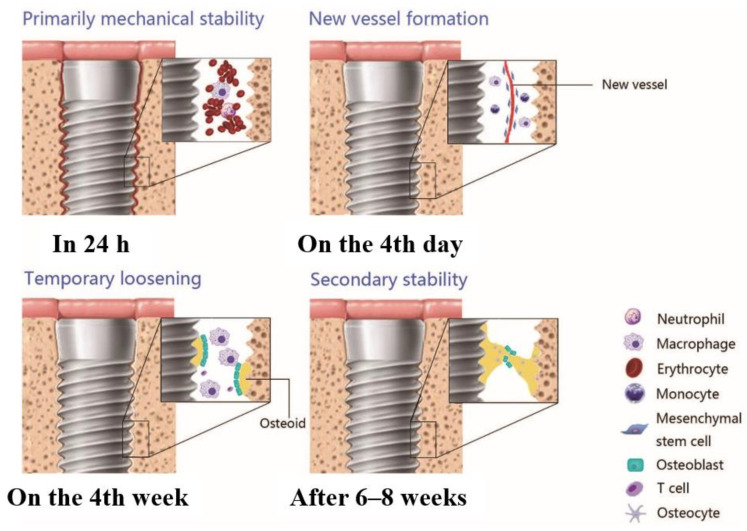
The process of osseointegration over time [18]. Reprinted with permission from Ref. [18]. 2015, John Wiley and Sons.

The research of osseointegration began in the 1950s when bioengineer Per-Ingvar Brandmark found that the titanium component could only be retrieved if the surrounding bone was destroyed after implanting titanium components into rabbit femurs [14]. Since then, titanium-based implants have been frequently adopted clinically due to their acceptable integration capabilities with the host bone tissue [15]. Osseointegration is the direct interaction of structure and function between the bone tissue and the implant’s surface in the early stages [16]. Through its in-depth study, osseointegration is now well-recognized as an active and spatiotemporal process that occurs throughout the healing process [17]. Briefly, the process goes through multiple stages, including blood clot formation, immune responses, angiogenesis, and osteogenesis (Figure 1).

### 2.2. Four Stages throughout the Osseointegration

#### 2.2.1. The Formation of Blood Clots

The implantation of the implant invariably results in a vascular rupture in the surrounding area. As a result, the implant’s surface contacts the patient’s blood. A blood clot immediately forms on the implant’s surface several hours after imploding [17]. The blood clot may last up to 4 days after an injury [19].

The most evident contribution of blood clot formation is to limit bleeding, after which the blood clots act as a temporary matrix for wound healing [19], which can speed up wound healing [20]. They also create cell-instructive microenvironments; for example, blood clots have been found to promote human marrow stromal cells’ (h MSC) proliferation, extracellular matrix remodeling, and the release of matrix fragments and angiogenic vascular endothelial growth factor (VEGF) [20].

#### 2.2.2. Immune Responses

During the first 24 h, the inflammatory process response peaks after the blood clot formation and lasts 7 days following the injury [21]. A favorable immune response can manipulate an osteogenic microenvironment, but an improper immune response might lead to persistent inflammation and the creation of a fibrous capsule surrounding the implant [22]. Immune cells contribute to the inflammatory response, including monocytes, macrophages, osteoclasts, leukocytes, and multinucleated giant cells [23]. The apparent significance of macrophages and their secrets have been emphasized recently. Macrophages are the most important effector cells in immune responses [24] and are required for osteogenesis. In a transgenic rat model of heterotopic ossification, osteogenesis was severely reduced when liposome-encapsulated bisphosphonate was delivered regionally to partially deplete tissue macrophages. The elimination of the BMP4 produced by macrophages in the injured area was responsible for this effect [25]. Bone regrowth in a fracture callus was wholly prevented in a bone fracture model when macrophages were reduced with liposomal clodronate [26].

Additionally, macrophages develop specialized and polarized functional characteristics in the setting of a particular immune response. Traditional activated macrophages (also called M1) and alternatively activated macrophages (also called M2) are two types of polarized macrophages [27]. IFN-γ can activate M1 macrophages alone or be combined with microbial stimuli or cytokines with a pro-inflammatory capacity [28]. IL-4 or IL-13, IL-10, glucocorticoid, or ketosteroid hormones are cytokines that can activate macrophages’ activity towards an M2 phenotype [29,30]. They aid in eliminating inflammation and further bone healing through a fulfilled osseointegration [31].

#### 2.2.3. Angiogenesis

The formation of new blood vessels is known as angiogenesis [32]. This process begins within two to three days of implantation [18]. New blood vessels are formed from the original vascular tissue. To make it easier for a capillary sprout to form and migrate to the damaged bone tissue, the basement membrane of an existing blood artery must be degraded. Various growth factors, such as VEGF and PDGF, attract endothelial progenitor cells to damaged sites. The epithelial progenitor cells migrate to newly formed vessels through circulation. Enzymes degrade proteins in the extracellular matrix near the capillary tips, which causes the vascular front to advance. Following the creation of vascular tubes and loops, the new channel is matured by recruiting smooth muscle cells and pericytes, which surround and maintain the blood’s capillary action and allow the blood to circulate [33]. Several growth factors, such as acidic and basic FGF, angiogenin, transforming growth factors α and β [34], and especially VEGF, play a pivotal role in angiogenesis [35].

Osteogenesis and angiogenesis are closely related since blood vessels transport nutrients and oxygen to organs during bone regeneration. In response to hypoxia, osteoblasts activate the hypoxia-inducible factor-α (HIF-α) pathway. HIF-α promotes angiogenesis and osteogenesis by increasing the VEGF levels in osteoblasts. The mice which were genetically modified by the deletion of the von Hippel–Lindau gene expressed large amounts of VEGF and developed extremely dense and well vascularized long bones [36]. In tissue-engineered structures, developing a vascular system promptly enhances cell survival and consequently facilitates bone regeneration.

#### 2.2.4. Osteogenesis

Mesenchymal cells, pre-osteoblasts, and osteoblasts link to the implant’s surface covered by the calcified fibrillar layer on the first day after implantation to generate collagen fibrils of osteoid tissue. A braided bone and later a reparative trabecular bone grow at the gap between the implant and the host bone after a few days of implantation, with bone trabeculae delimiting vast marrow areas rich in blood vessels and mesenchymal cells [37]. The osteoblast is a critical effector cell in the process of osteogenesis. The word “osteoblast” was coined in the early twentieth century to describe bone-forming cells [38]. Due to their multipotency, MSCs are distinguished from osteoblasts. This process is directed by so-called “master transcriptional regulators”, with Runx2 serving as the master transcriptional regulator for the osteoblast lineage. Collagen type 1α1, osteocalcin (OC), and alkaline phosphatase (Alp) are among the bone matrix proteins secreted by osteoblasts [39].

Bone formation, modeling, and remodeling comprise the three processes during osseointegration. Osteoblasts and bone-resorbing osteoclasts collaborate closely during these processes, forming a “bone multicellular unit” [40]. Osteoblasts drive the extracellular matrix’s formation, while osteoclasts cut it out to fit the physical environment (modeling) and adapt it to the demands of body growth and changing settings (remodeling).

## 3. The Effect of the Nervous System on Bone

According to data from clinical and experimental investigations, the neurological system, including neurotrophins, neuropeptides, and nerve cells, appears to have a role in diverse bone functions. A paucity or complete loss of peripheral innervation might result in a fracture without the bone healing [41]. The callus development and frequency of the union of nailed femur fractures in patients with accompanying head traumas and likewise treated fractures without head injuries were compared in research. The study revealed that fractures in individuals with head traumas recover with increased calluses and faster than usual [42]. Another study investigated how neurologic involvement affected bone mineralization. They demonstrated that when neurologic involvement increases, the volume and density of the bone tissue decrease, and weight-bearing may modestly mitigate this effect [43]. Moreover, after unmyelinated sensory neurons in rats were specifically lesioned with capsaicin, the trabecular integrity was lost, and neuropeptides in the nerves and bone were depleted [44].

### 3.1. Neurotrophins (NTs) and Their Receptors

Nerve growth factor (NGF), brain-derived neurotrophic factor (BDNF), neurotrophin-3 (NT-3), and neurotrophin-4 (NT-4) are neurotrophins (NTs). They share a high level of similarity and trigger biological reactions that are very comparable. Neurotrophins are generated by non-neuronal cells such as leukocytes and fibroblasts and act on non-neuronal cells in various ways. The growing amount of evidence implies that NTs play an important role in bone formation [45]. Neurotrophins have biological effects by activating both high-affinity and low-affinity receptors. TrkA, TrkB, and TrkC are tyrosine kinase receptors with a high affinity for tyrosine, while p75 has a low affinity.

Furthermore, all neurotrophins target p75, a tumor necrosis factor receptor family member [46]. TrkA was discovered to be a necessary receptor for transmitting physiological responses to NGF. Since human Trk was transfected into NGF-nonresponsive PC12 cells missing the natural protein, a wide range of biological reactions to NGF were restored [47].

The receptor’s dimers and the kinase are activated when a neurotrophins bind to Trk receptors. Each Trk receptor has ten conserved tyrosines in its cytoplasmic domain, three of which are found in the kinase domain’s autoregulatory loop. The phosphorylation of these amino acids further activates the kinase. The other residues’ phosphorylation aids signaling by establishing docking sites for adaptor proteins that connect these receptors to intracellular signaling pathways such as the Ras/ERK protein kinase pathway, the PI3 K/Akt kinase pathway, and the PLC-γ1 pathway [48,49].

P75 binds to the whole neurotrophin family. It belongs to the TNF receptor/Fas/CD40 superfamily of receptors and is a transmembrane glycoprotein. The p75 receptor can alter neurotrophin activities by interacting with various Trk receptors [50]. The high-affinity binding of NGF is increased when p75 and TrkA are co-expressed [51]. These findings show that the p75 receptor may work with other Trk receptors to alter ligand selectivity, binding, intracellular transport, and signal transduction. p75 has different roles when produced in the absence of a Trk, in addition to its capacity to influence the binding and function of Trk receptors. In the absence of an alert Trk receptor, p75 expression in neuronal cell lines has been shown to induce cell death [52]. The activation of p75 signaling might be a critical factor in neuronal death regulation. The cytoplasmic effectors receptor-interacting protein 2 [53] and NRIF [54] are recruited and released by p75 through ligand-mediated recruitment and release. Several of the critical downstream signaling pathways induced by p75 in reaction to neurotrophins include NF-B [55], Jun kinase (JNK) [56], and caspases [57].

Table 1 summarizes the receptors and effect of neurotrophins on bone.

#### 3.1.1. NGF

Rita Levi-Montalcini found NGF in the 1940s, which was isolated in high quantities by the 1960s [58]. The high-affinity NGF receptor is TrkA, while the low-affinity NGF receptor is p75. Rat BMSC and human monocytes also express the NGF-specific receptor TrkA [59]. NGF has been found in a variety of cells in several investigations. In embryonic chick skeletal tissue, NGF has been found in chondrocytes and osteoblasts. According to the findings, NGF determines the sympathetic innervation density in developing skeletal tissues [89]. On the other hand, another research study found no NGF expressed in chondrocytes. This situation might imply that NGF emerges during chondrocyte differentiation at a certain point in callus formation. NGF was found in skeletal cells and the matrix of both fractured and unfractured adult rat bones [90]. The expression of NGF mRNA in the broken rib grew during the recovery process, then peaked two days following the fracture [64].

NGF is required for bones to respond to mechanical stress. The surfaces of mammalian bones are extensively innervated by sensory neurons that emerge from the dorsal root. Mechanical impulses cause osteoblasts on the bones’ surface to produce more NGF, which stimulates TrkA sensory neurons, causing the release of osteogenic cues that govern osteocytic Wnt/-catenin signaling and promote bone formation. The osteocyte is the main bone cell in charge of translating mechanical stimuli into signals that affects bone turnover. These cells have extensive dendritic links to bone cells throughout the skeleton and the capacity to produce both anabolic and catabolic signaling molecules, suggesting that they might serve as mechanosensors [60].

The ability of topically given NGF to enhance bone formation was tested using a rat model of mandibular distraction osteogenesis. It was discovered that NGF may enhance innervation and bone regeneration and induce osteoblastic cell differentiation [61]. As shown by the increased cell proliferation, higher ALP activity, and up-regulated expression of osteogenesis-related genes and proteins, BCP coupled with NGF had a powerful effect on osteoblast differentiation [62]. NGF stimulates the differentiation and activation of osteoclasts [63]. Through the control of osteogenesis and bone resorption, NGF directly impacts bone repair and remodeling during osseointegration.

Treatment with anti-NGF antibodies significantly reduces the pain behavior associated with bone cancer. Using a prostate-specific bone cancer model in a mouse femur, NGF-sequestering antibodies were administered. It was observed that the administration of a blocking antibody to NGF significantly reduced both early- and late-stage bone cancer pain-related behaviors to a level equivalent or greater to that observed following an acute administration of 10 mg/kg or 30 mg/kg of morphine sulfate [91].

#### 3.1.2. NT-3

NT-3, a neurotrophin, plays a pivotal role in spinal neurons’ survival and the regeneration of axons, embryonic blood vessels, limb bone development, vascular endothelial cell migration, and other processes [92]. NT-3 and its particular receptor TrkC are both expressed by human monocytes. NT-3 has been shown to aid in the repair of bone fractures. NT-3 mRNA expression increased in a damaged ribcage throughout the repair procedure, peaking two days after the fracture. At the fracture callus, NT-3 was discovered in osteoblast-like cells and hypertrophic chondrocytes [64]. NT-3 increases the formation of osteoblasts, which aids in bone fracture repair. NT-3 promotes the differentiation of rat BMSCs into osteoblasts while blocking adipocyte differentiation [65], both directly (by activating Erk1/2 and substantiating RANKL-induced enhanced expression of osteoclastogenic signals in developing osteoclasts) and indirectly (by enhancing osteoclastogenesis and resorption, and by inducing osteoclastogenic signals in osteoblasts) [66]. Additionally, by the modulation of the endothelial–mesenchymal transition (EndMT), NT-3 may increase the production of heterotopic ossification. Furthermore, via activating EndMT, NT-3 increased the expression of achondrogenesis markers and osteogenesis indicators (OCN and Runx2) [67].

#### 3.1.3. NT-4/5

TrkB is a single high-affinity receptor that NT-4/5 binds to and activates. The granulocytes produced mRNA for the NT-4/5 gene [68]. TrkB positivity was found in eosinophilic metamyelocytes and polymorphonuclear cells. Shortened TrkB expression was identified in erythroblasts and megakaryocytes [69]. During tooth development, the expression of NT-4/5 can be seen [70]. Through the ERK1/2 signaling pathway, NT-4/5 modulates the activities of periodontal ligament cells and stimulates the mRNA production of ALPase, OPN, and BMP-2 in HPL cells [71,72].

#### 3.1.4. BDNF

Brain-derived neurotrophic factor (BDNF) was identified in the 1980s and is found in trace amounts in the central nervous system [93]. BDNF has its own specific receptor: TrkB. BDNF has been discovered to favorably control new bone production by promoting osteoblast growth, differentiation, and mineralization [73]. BDNF increases the proliferation and differentiation of mesenchymal stem cells into osteoblasts [94]. BDNF boosted the differentiation of MC3T3-E1 cells and accelerated new bone production and maturity according to research into the influence of BDNF on osteogenesis [74].

Moreover, BDNF plays a key role in the activation of osteoclasts. Research on multiple myeloma (MM) discovered that human osteoclast precursors expressed BDNF receptor TrkB and a Trk inhibitor, K252a, significantly decreased BDNF-stimulated osteoclast production, suggesting that BDNF utilized TrkB for its influence on osteoclasts. BDNF increased the expression of the RANKL in bone marrow stromal cells, promoting osteoclasts’ development [75]. Polymorphisms in the BDNF gene have been related to an increased risk of distal radius fractures and BMD. In a retrospective study of 152 participants with distal radius fractures and 148 control subjects, the BDNF polymorphism rs6265 increased the risk of osteoporosis and distal radius fractures [76].

#### 3.1.5. GDNF

Glial cell line-derived neurotrophic factor (GDNF) was initially identified as a glioma cell line-secreted factor that might help fetal ventral midbrain neurons survive in vitro [95]. RET, GFRα-1, and GFRα-2 are all GDNF receptors [78]. GDNF can regulate bone metabolism. TNF-stimulated MC3T3-E1 cell proliferation was boosted synergistically by GDNF, indicating that GDNF seemed to engage in TNF-induced activation in osteoblastic cells [79]. A neutralizing antibody against GDNF reduced BMMSC migration in a pre-osteoclast-conditioned media, while therapy with recombinant GDNF increased migration and osteogenic differentiation [80]. Keijo Luukko and colleagues discovered that GDNF works as a neurotrophic factor generated from a target during dental innervation [81].

#### 3.1.6. PDGF

Platelet-derived growth factors (PDGFs) are important mitogens for numerous mesenchymal cell sorts, such as fibroblasts and smooth muscle cells, as well as certain neuroectodermal cell populations, such as oligodendrocytes. PDGF has many variants, each communicating with two unique dimerized receptors (A and B) that have varied affinities [83]. According to cross-competition studies, the type A PDGF receptor bound to all three dimeric forms of PDGF. However, the type B PDGF receptor bound to PDGF-BB with a high affinity and PDGF-AB with a reduced affinity but did not bind to PDGF-AA [84].

During osseous repair, PDGF is among the most essential biological agents. PDGF’s capacity to serve as a chemoattractant and mitogen for mesenchymal cells causes angiogenesis and recruits osteoprogenitor cells to contribute to its powerful stimulatory actions [85]. PDGF is released initially by degranulating platelets in a broken hematoma. Macrophages travel into the fracture site in response to the break stress and the first production of PDGF by platelets and further express PDGF [86]. In vitro, PDGF stimulates osteoblasts and acts as a mitogen and chemotactic factor. During wound healing, it stimulates bone formation in vivo. The migration of osteoblastic cells was stimulated by PDGF, but proliferation was not affected [96]. In vitro mature osteoclasts control osteoblast chemotaxis via PDGF-bb/PDGFR-β signaling [97]. According to a study, zoledronate suppressed angiogenesis and osteogenesis by suppressing preosteoclasts releasing PDGF-BB [98].

#### 3.1.7. FGF

The fibroblast growth factor (FGF) gene family now has nine members, all of which are expressed in mammals. Acidic and basic FGF, commonly known as FGF-1 (aFGF) and FGF-2 (bFGF), are the most prevalent in normal adult tissues. To transduce signals in target cells, FGFs bind to numerous FGFRs. A mouse fracture model investigated the influence of an aFGF injection on normal wound repair. The aFGF infusions boosted cartilage size while decreasing type II procollagen and proteoglycan-essential protein mRNA expression [87]. FGF-2 has been detected in the upper hypertrophic areas of the growth plate, suggesting its function in chondrocyte development and endochondral bone formation. FGF-2 induces bone resorption in addition to its mitogenic and angiogenic characteristics. Based on these characteristics, FGF-2 has the potential to influence different stages of fracture recovery, from early post-traumatic events to late callus reformation. In developing rats, a modest dosage of bFGF increases endochondral bone development while suppressing periosteal bone growth [88].

### 3.2. Neuropeptides

Neuropeptides are small proteinaceous molecules generated by nerves, which are produced at nerve terminals upon neuron activation, and then bind to neural sensors to operate on neuronal substrates [99]. The data suggest that neuroendocrine factors influence bone physiology [100]. The neuropeptide-containing nerve changes in various clinical-experimental instances imply that they are involved in local clinical conditions such as bone formation, healing, and restructuring (Table 2). Furthermore, patients with neurologic problems have been demonstrated to have localized bone alterations, altered fracture healing, and increased callus development in clinical circumstances [12].

#### 3.2.1. CGRP

Calcitonin gene-related peptide (CGRP) is a peptide that belongs to the calcitonin family. The whole nervous system, the cardiovascular and respiratory systems, and the thyroid gland all contain CGRP. Bone marrow and periosteal tissues were similarly shown to have an equal distribution of CGRP [127]. Humans produce two types of CGRP: α-and β-CGRP. CGRP targets a variety of non-neuronal tissues, including bone.

CGRP has been found to aid in transmitting pain and sensitization [101]. In osteoarthritis, the function of CGRP in musculoskeletal pain has been extensively characterized. Serum CGRP levels have also been positively linked with pain severity [102]. Since CGRP is widely distributed in fracture tissue, it is expected to have a role in fracture pain. More research has been conducted on the involvement of CGRP in osteoclasts and bone resorption. CGRP receptors were found on the plasma membrane of BMMs and osteoclasts by Wang et al. In BMMs, CGRP inhibits osteoclastogenesis and bone resorption via blocking the RANKL-mediated activation of NF-κB [103,104]. These findings showed that CGRP might limit bone resorption by regulating osteoclast development and activity directly.

CGRP promotes bone growth and inhibits bone resorption. CGRP has been shown to trigger various cell types’ differentiation and culturing into mature osteoblasts by many researchers. Examples include the proliferation of BMSCs that was stimulated by CGRP, which induced osteoblastic gene expression [103], and adipose-derived stem cells on a 3D calcium alginate gel [105]. Complete CGRP receptors have been found on BMSCs and osteoblasts’ plasma membranes. CGRP stimulates the expression of mature osteoblast genes. CGRP^+^ neurons are related to initiating bone growth, accelerated bone formation, and cartilage and muscle specialization in limb bud development [106,107]. As a result, CGRP^+^ neurons have been linked to the regulation of local bone formation.

CGRP^+^ nerves are important for fracture healing. According to Li et al., the overall data suggest that a fracture causes an intense, localized in-growth of new sensory neurons containing CGRP, which might be a requirement for wound repair and modeling [108]. In addition to functioning as a barrier against excessive fracture movement, CGRP-containing sensory innervation may play a key role in injuries’ vascular control, angiogenesis, and osteogenesis [109]. These investigations show that CGRP^+^ sensory neurons can facilitate fracture healing throughout the bone regeneration phase.

A few researchers have clarified CGRP’s involvement in fracture healing. NOS-mediated vasodilation is one mechanism by which CGRP helps in fracture healing. During bone healing, NOS expression and activity show a favorable connection with CGRP expression [110]. An immunofluorescent technique was used to study the healing of an experimental bony defect in a rat’s tibia. An increase in immunofluorescent nerve fibers of SP and CGRP was observed in the experimental tibia from the 6th day after the operation until the 24th day when they reverted to normal. Most of the SP- and CGRP-immunofluorescence was seen near the vessels. The nerves appeared to contribute to callus formation by enhancing the local blood flow [114]. In addition to mediating vasodilation, NO is a secondary messenger that aids in inflammation, vascularization, and other pathways throughout bone repair [111]. Local CGRP can also speed up fracture healing by stimulating osteogenesis. Research into the healing of fractures in rats showed the proliferation of nerve fibers containing the sensory neuropeptide CGRP within and around the callus [128]. CGRP inhibitors may prevent fracture healing by inhibiting the phosphorylation of ERK [112].

#### 3.2.2. SP

Substance P (SP) is a tachykinin-like peptide of 11 amino acids [129]. Von Euler and Gaddum first detected it in horse brain and stomach extracts capable of causing hypotension and muscular contractions in 1931 [130]. It acts on the neurokinin-1 receptor to initiate signal transduction cascades (NK1-R). Non-neuronal cells can create SP and can function through it. SP^+^ nerves have been found in the periosteum, subchondral bone, bone marrow, ligaments, and synovium, among other places in bone tissue [100].

A recent study has demonstrated that SP may affect fracture healing. The repair of a bone lesion in a mouse’s tibia has been investigated to determine when and where SP might appear. Since most of the SP-immunofluorescence was seen near the arteries, it was determined that the SP-immunofluorescent nerves collaborated in the callus development by increasing the local blood flow [114].

The metabolism of bones is affected by SP. The osteoporotic consequences of sciatic neurectomy are exacerbated by a broad decrease in substance P concentration in bone [115]. Besides its direct impact on bone cells, SP impacts bone metabolism through its effects on blood vessels and the generation of other cytokines. The increase of Ca^2+^ by SP may drive the remodeling of bone by osteoclasts [116]. Through an increased expression of RANKL, SP in the joint cavity may enable both synovial enlargement and the induction of enhanced osteoclast production [117]. SP can also improve bone resorption by increasing the synthesis of PGE2 and RANKL [118].

#### 3.2.3. VIP

Vasoactive intestinal peptide (VIP) has been proven to be a 28-amino-acid pre-pro-VIP cleavage product [131]. VIP^+^ nerves are commonly found in a bone’s epiphysis and periosteum [132]. VPAC1, VPAC2, and PAC1 are three VIP receptor subtypes [133]. By reducing bone resorption, VIP treatment aided bone-fracture repair. In sympathectomized mice, a VIP treatment significantly improved the bone density and mechanical features while enhancing the osteogenesis marker expression, reversing the inhibitory activity of 6-hydroxydopamine towards bone formation [119]. VIP can stimulate osteoblastic activity by promoting ALP and increasing calcium accumulation in bone nodules [120,121]; meanwhile, VIP decreases osteoclastic activity by inhibiting osteoclast formation [122].

#### 3.2.4. PACAP

Pre-pro-VIP is used to make pituitary adenylate cyclase-activating peptide (PACAP). PAC1, VPAC1, and VPAC2 are three kinds of receptors of PACAP. PACAP has the most vital attraction to PAC1, while the latter is similarly drawn to PACAP and VIP [134]. In preclinical OA models, PACAP was found to have anti-inflammatory and chondroprotective properties. After an OA induction, the PACAP levels in injured cartilage and SF were also lowered. PACAP inhibited IL-1-induced chondrocyte death in vitro. The reduced i-NOS and COX-2 levels paralleled these alterations, indicating an anti-inflammatory action [123].

#### 3.2.5. NPY

Tatemoto et al. were the first to isolate Neuropeptide Y (NPY) from pig brains [135]. The five Y receptors that have been recreated in mammals so far are Y1, Y2, Y4, Y5, and Y6 [136]. There is evidence suggesting that NPY may regulate bone homeostasis. NPY is expressed in bone cells. At both the embryonic and adult stages, osteoblasts, osteoclasts, and chondrocytes synthesize NPY. It was observed that transthyretin KO mice had an increased bone mineral density and trabecular volume owing to higher levels of amidated neuropeptide. Not only is NPY expressed in MC3T3-E1 osteoblastic cells and BMSCs, but it can also be detected in BMSCs in the process of osteoblast differentiation [137]. In osteoprogenitor cells stimulated by NPY, osteoblast markers are significantly elevated, probably due to a reduced Y1 receptor expression. In contrast, it is most likely that Y2 receptor signaling is responsible for reducing the calcium deposition in the extracellular matrix in these cells. Moreover, at the early stages of osteoblast differentiation, NPY inhibits the transcriptional activity of the RANKL promoter in osteoprogenitor cells and enhances the expression of OPG [124]. There is some indication that the NPY system can also control bone resorption. NPY inhibited isoprenaline-induced osteoclastogenesis in rat BMCs by inhibiting agonist-induced increases in cAMP and RANKL production [125]. At the early phases of differentiation, NPY reduces the transcriptional activity of the RANKL promoter in osteoprogenitor cells and increases OPG expression in osteoblasts. When acting directly on osteoblasts, the NPY pathway aids in bone homeostasis [124].

### 3.3. Nerve Cell

#### 3.3.1. Schwann Cell

**Figure 2:**
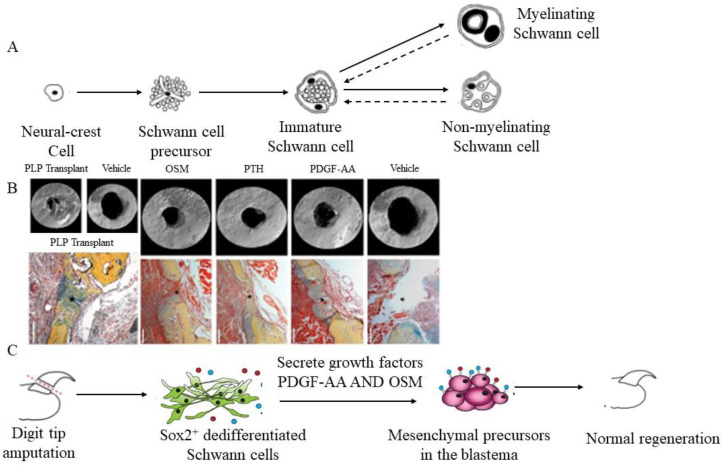
(**A**): The main stages in the development of Schwann cells [138]. (**B**): Schwann cells perform a critical function in bone tissue repair. The mTomato-positive Schwann cells from PLP-treated mandibles formed more bone than the vehicle-treated mandibles [140]. (**C**): Nerve-associated DSCs secrete paracrine substances to help the distal digit of a rat regenerate [142]. Reprinted with permission from Ref. [142]. 2017, Elsevier.

The Schwann cell is one of the most common cell types in the nervous system. The neural crest generates the vast majority of Schwann cells in the peripheral nerves of mammals (Figure 2A). Endothelins govern the existence of Schwann cell progenitors in Schwann cells in vitro and in vivo [138]. MSCs can grow into Schwann cells when exposed to pre-inducing chemicals such as BME and RA, accompanied by inducing factors including bFGF, PDGF, and heregulin (HRG) [139]. A recent study demonstrated that functional abnormalities in mouse skeletal stem cells (mSSC) limit mandibular bone healing following an inferior alveolar denervation. The fundamental mechanism of mSSCs is paracrine substances produced by Schwann cells, with Schwann cell regeneration and Schwann-derived growth factors partly rescuing the denervated phenotype (Figure 2B) [140]. Schwann cell precursors are involved in generating mesenchymal and osteoprogenitor cells in mice and the embryonic development of zebrafish [141].

Additionally, Schwann cells perform a critical function in bone tissue repair (Figure 2C) [142]. Schwann cells are found in damaged periodontal tissue, indicating that the cells play an essential role in wound healing, particularly in alveolar bone tissue [143]. Nerve-associated SCPs dedifferentiate after the distal digit of a rat is removed. Dedifferentiated Schwann cells (DSCs) secrete paracrine substances that help them regenerate. Following digit amputation, DSCs significantly enhance PDGF-AA and oncostatin M (OSM) mRNA in vivo and release both ligands under culture conditions [144]. OSM has pleiotropic roles in bone metabolism and homeostasis, whereas PDGF-AA is a potent therapeutic agent for bone growth and repair [145,146]. Mesenchymal precursor proliferation in the blastema was reduced and nail and bone regeneration was hindered when SCPs were abnormally regulated or ablated. Exogenous SCPs were transplanted to help with these regeneration issues [144].

Osteoblast growth and differentiation are aided by Schwann cells. Co-culturing osteoblasts with Schwann cells can increase their proliferation and differentiation. Another research study reported that in an in vitro indirect coculture setting, Schwann cells preserve their regular potential to generate neurotrophins, boosting osteoblast proliferation and differentiation [73]. Semaphorin (Sema) 3A, a chemorepellent protein, helps regulate bone mass. The interactions between Schwann cells and Sema3A occur under diverse conditions. In a single cell culture, Sema3A inhibits the osteogenic differentiation of MG63 cells and promotes it in coculture with Schwann cells in a concentration-dependent manner. These data indicate that Schwann cells are able to induce osteogenic differentiation in bone cells by acting on Sema3A [147].

#### 3.3.2. Sensory Nerve Cell

The quality of the new bone generated throughout mandibular distraction osteogenesis may suffer due to the lack of sensory nerves [148]. Sensory neurons sensitive to capsaicin are crucial in bone modeling [149]. In a recent study, capsaicin was used to specifically destroy non-myelinated sensory neurons in mice. After the induction of neuronal lesioning, the trabecular integrity was lost, the bone mass was reduced, and the neuropeptides in the nervous system and bone were depleted. These findings imply that sensory neurons sensitive to capsaicin serve a function in preserving trabecular bone integrity [44]. Study results have demonstrated that Sema3A modulates sensory nerve development indirectly to regulate bone remodeling. Due to a decrease in bone formation, Sema3a^−/−^ mice have a low bone mass. Even though bone-specific Sema3A-deficient mice had a reduced expression of Sema3A, their bone masses were normal. On the other hand, mice lacking Sema3A in their neurons fell short in terms of bone mass, similar to Sema3a^−/−^ mice, indicating that neuron-derived Sema3A accounts for the observed abnormalities in bone [150].

Sensory neurons can affect the development of osteopenia. Prostaglandin E2 (PGE2) produces bone pain by sensitizing DRG neurons via altering the voltage-gated sodium channel NaV1.8 [151]. Sensory neurons can crosstalk with cancer cells, which contributes to bone discomfort. Blocking sensory nerve stimulation may have anti-cancer benefits towards bone metastases and analgesic impacts on bone pain [152].

Many studies have reported the existence of sensory neurons that innervate bone. Sensory neurons innervate the periosteum and the bone marrow. The periosteum is innervated both by large diameter, fast conducting units with encapsulated endings and by small diameter, slower conducting units with free fiber-endings typical of nociceptors. Both fast, sharp, and slow-burning bone pain may be caused by their response properties [153]. Physiological saline was injected under pressure into the bone marrow cavity to observe the reaction in dogs. The injections resulted in strong pain-like behaviors in the dogs [154,155].

## 4. The Impact of the Nervous System on Each Stage of Osseointegration

### 4.1. Immune Responses

Monocytes [156], mastocytes [157], and B or T cell clones [158] have been shown to have functional NGF receptors. NGF causes monocyte cytotoxicity, basophilic cell differentiation, mast cell formation, and degranulation, among other pleiotropic actions [69]. Following stressful circumstances, NGF functions as a broad “alert” molecule that may recruit and prime both local and systemic defensive systems. In methylcellulose hematopoietic colony tests of human peripheral blood, NGF stimulated human granulopoiesis, significantly increasing basophilic cell development [159].

GDNF, and to a lesser extent, NT-3, can activate the immune system in allogeneic graft combinations. However, these trophic factors do not produce overt rejection or immune responses [160].

BDNF aids the inflammatory response. Microglia, the central nervous system’s primary innate immunity cells, can release it, and their actions may be influenced by BDNF [161]. Human BDNF’s genetic variants may alter macrophage phenotype, according to research. In the infarcted heart of mice expressing the human BDNF Val66Met polymorphism, there was a sustained presence of pro-inflammatory M1-like macrophages and a decreased accumulation of reparative-like phenotypic macrophages. Compared to wild-type peritoneal macrophages, the qPCR analysis revealed that BDNF^Met/Met^ peritoneal macrophages are more pro-inflammatory and have a higher migratory potential [162].

FGF-21 may have a function in immunoregulation and anti-inflammation. Exogenous FGF-21 treatment can attenuate inflammation produced by lipopolysaccharides in vivo. FGF-21 inhibited LPS-induced IL-1 expression in THP-1 cells in vitro. Furthermore, FGF-21’s mechanism of action was discovered to include the increased IL-10 in the ERK1/2 and NF-κB pathways [163]. FGF-21 may influence the immune response indirectly by altering immune cell metabolism. FGF-21 increases GLUT-1 expression, which controls glucose absorption. Glucose is a significant energy source for monocytes, which play a key part in the innate immune system of the human body [164].

CGRP promotes a powerful peptidergic anti-inflammatory mechanism in neuro–immunological interactions. CGRP suppresses macrophages and dendritic cells’ ability to create inflammatory cytokines and deliver antigens to T cells by acting directly on them. Some of the effector mechanisms by which CGRP acts on innate immune cells include the overexpression of IL-10 and repression of NF-B activity [165].

SP is a key regulator of cellular motility, functioning explicitly or implicitly through synthesizing chemokines and adhesion molecules. SP induces IL-8 production-a strong neutrophil chemotactic protein—in human corneal [166] and epithelial cells [167], neutrophils [168], mast cells [169], and fibroblasts. SP promotes the growth of immunological cells. T cell proliferation is influenced by SP [170]. SP stimulates the proliferation of mononuclear cells and fibroblasts in human bone marrow [171]. By influencing the activation of innate immune cells such as natural killer cells, macrophages, mast cells, and eosinophils, SP enhances their survival.

VIP and PACAP reduce activation-induced cell death in peripheral T cells and T cell hybridomas in vivo and in vitro. The VPAC1 and VPAC2 receptors mediate the effect, which is dose-dependent [172].

### 4.2. Angiogenesis

NGF has a function in postnatal angiogenesis. NGF’s proliferative impact on human dermal microvascular endothelial cells was initially documented by Raychaudhuri et al. in 2001. NGF is a chemoattractant for EC that can cause human and pig aortic EC to migrate [173,174]. In the rat cornea, NGF induces a dose-dependent angiogenic response [175]. Anti-VEGF-A antibodies inhibit NGF-induced CAM neovascularization, suggesting that VEGF-A is involved in NGF-induced angiogenesis [176,177].

Some of NGF’s angiogenesis-related functions were later shown to be shared by BDNF. Similar to NGF, BDNF is thought to upregulate VEGF-A expression through the TrkB pathway. The ischemia limb mouse model investigates the possibility of using BDNF as a gene therapy for angiogenesis. Gene transfer-induced BDNF overexpression increases the vascular density of ischemic muscle, hastening blood circulation recovery [178]. Unlike NGF, BDNF’s proangiogenic effects are not inhibited by VEGF neutralization. Following a myocardial infarction, BDNF enhances the angiogenesis caused by bFGF and improves heart function [179].

In the Matrigel model, an additional ligand of TrkB, NT-4/5, can also stimulate angiogenesis [178]. NT-3 was shown to have proangiogenic properties in several investigations. In human bone marrow mesenchymal stem cells, NT-3 greatly increased the release of VEGF, NGF, and other vasoactive substances in vitro [180]. The synergistic impact of NT-3 and VEGF on angiogenesis, anti-inflammation, and brain healing is beneficial [180]. In rats’ hind limbs, NT-3 overexpression increased muscle capillary and arteriolar densities and enhanced postischemic blood circulation recovery in the absence or presence of ischemia [181].

In normal skin, GDNF can mildly control the microvascular network [182]. Through human umbilical vein endothelial cells, it has been revealed that GDNF, alone or in an ASC-conditioned medium, promoted vascular network growth and that this effect was independent of VEGF activity [183]. Using both in vitro and in vivo modeling methodologies, GDNF’s activity was demonstrated to influence VEGF-driven angiogenesis, modifying endothelial cell sprouting and blood vessel growth [184].

Primary and stable endothelial cells respond to FGF and VEGF by surviving, proliferating, migrating, and differentiating [185]. The concurrent intravitreal release of both VEGF and bFGF causes retinal vascular alterations that lead to bleeding in rabbits [186].

CGRP has also been discovered to be a powerful vasodilator and inducer of angiogenesis [187]. Since CGRP relaxes rats’ aorta strips in vitro, it might be involved in blood flow control. The link between significant CGRP-containing fiber sprouting and necrotic endosteal bone remodeling suggests that CGRP-containing nerve neurons are crucial in angiogenesis control [188]. In vivo, CGRP induces angiogenesis via activating CGRP receptors [187].

The autocrine controller of the microvascular events required for neovascularization, NO, is produced by SP and mediates angiogenesis [189]. It has been established that SP is present in a specific subset of primary sensory neurons. In response to corneal inflammation, sensory neurons directly stimulate angiogenesis via SP signaling [190]. To enable the regulated release of SP in vivo, a biodegradable hydrogel was made from an anionic derivative of gelatin. The controlled delivery of SP stimulated the formation of circulating cells with angiogenic activity from the blood flow into the implanted area, causing increased angiogenesis [191].

### 4.3. Osteogenesis

**Figure 3:**
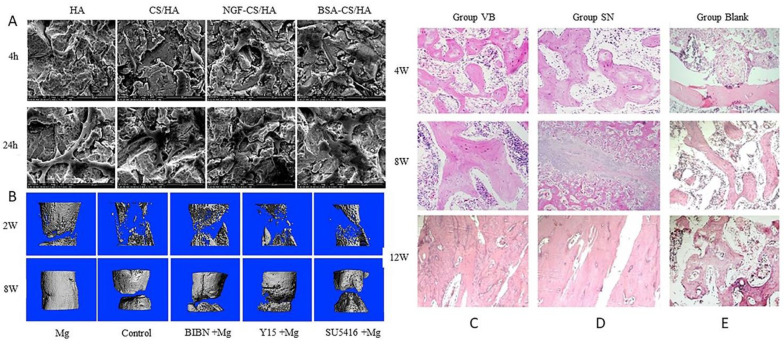
How the nervous system enhances the effect of osteogenesis, with examples from the literature. (**A**): NGF-CS/HA composite coating has positive impact on the behavior of BMSCs. 4 and 24 h after BMSC culture, each group had excellent adhesion and was triangular or symmetrical; BMSCs have spread over the coating surface and displayed protruding pseudopodia [192]. Magnification, ×2400. (**B**): A 3D image of newly formed bone in the defect gap in the Mg group showed greater mineralization than that in the inhibitor/antagonist +Mg group at both 2 and 8 weeks as compared with the inhibitor/antagonist +Mg group [201]. (**C**–**E**): The formations of new bone in group VB (**C**) and group SN (**D**) were better than that in group Blank (**E**) and there was no obvious difference between VB and SN at 4, 8, and 12 weeks (HE × 100) [204]. Reprinted with permission from Ref. [192]. 2020, Elsevier; Ref. [201]. 2021, Elsevier.

In BMSCs, the expression of the genes and proteins associated with osteogenesis and neural development was enhanced in NGF-CS/HA-coated combination titanium. NGF-CS/HA-coated composite titanium greatly aids the growth of BMSCs into osteoblasts and neural cells (Figure 3A) [192].

To increase bone formation during tissue engineering, BDNF may promote neurogenesis and osteogenesis in hBMSCs [193]. Peripheral nerve damage causes local BDNF expression, which speeds up sclerotic alterations in the alveolar bone. BDNF boosted new bone production and maturation in MC3T3-E1 cells through stimulating differentiation [74].

The heat created during an implant osteotomy may cause osteonecrosis and delayed bone healing, thereby compromising initial bone-anchoring osseointegration and fixation equipment. Core–shell polymeric biodegradable microspheres containing PDGF can avoid heat-induced bone damage. In one study, PDGF increased cell viability and decreased osteonecrosis, while PDGF-simvastatin administration in a sequential manner increased osteogenesis and encouraged bone maturation [194]. Magnesium stimulates MC3T3-E1 to secrete PDGF-BB, improving the osteoblasts’ differentiation into osteoblasts and facilitating HUVECs to be more angiogenic [195]. PDGF-B can stimulate the repair of missing oral implant sockets by gene delivery. Adenoviral vectors (Ad) encoding PDGF-B, luciferase, or recombinant human PDGF-BB protein were used to repair alveolar ridge defects created by titanium implant placement in rats. A comparison of Ad-PDGF-B and rhPDGF-BB delivery to Ad-Luc demonstrated that bone repair was enhanced by Ad-PDGF-B and rhPDGF-BB, with the high dose of Ad-PDGF-B providing greater benefit [196].

Moreover, BMSCs transfected with bFGF will significantly enhance vascular tissue repair and increase osseous formation and remodeling [197]. The effects of recombinant human FGF-2 and melatonin on the osteogenesis surrounding titanium implants were investigated. In the experimental group, newly produced bone was found around the titanium implant. In addition, the medullary canal region contained an abundance of bone trabeculae, which was not the case in the comparison group. Melatonin and FGF-2 possess the capability to induce osseointegration [198].

CGRP may promote osseointegration in the early to middle stages since it modulates peri-implant angiogenesis and osseointegration dysfunctions caused by diabetes [199]. The IAN titanium implant-bone model revealed a CGRP deficit and decreased osteogenesis around the implant [200]. Mg implants have a significant potential for shortening DO treatment durations in practical applications, and when Mg is combined with DO, a CGRP-FAK-VEGF signaling axis linking sensory neuron and endothelial cells may be the main mechanism behind critical size bone defect repair Figure 3B [201].

The sensitivity for loads is relatively modest in the situation of the implant. Grafting Schwann cells into the implant site has been shown to increase the sensory reactions of dental implants to a rate equivalent to natural teeth. After a peripheral nerve injury, Schwann cells form a cellular ring to accept regenerated sprouts from the axonal stump. Secondly, it may produce several neurotrophic factors and receptors, including NGF, BDNF, NT-3, and GDNF, which are necessary for axonal regeneration after nerve injury [202]. Schwann cells may also generate surface cell adhesion molecules such as N-CAM and L2/HNK-1, as well as an extensive basement membrane comprising laminin, HSP, and tenascin, among other extracellular matrix proteins. The factors promote axon extension by enhancing the “adhesiveness” of the substrate, enabling axon-to-axon and axon-to-Schwann cell connections. Therefore, Schwann cells might be useful for enhancing nerve regeneration in the peri-implant environment [203].

It has been documented that sensory nerves play a role in osteogenesis. When sensory nerve tracts are implanted into tissue-engineered bone, vascularization and neurotization are greatly improved, resulting in a greater osteogenesis impact than tissue-engineered bone alone (Figure 3C–E) [204].

## 5. Future Outlook and Limitations

**Figure 4:**
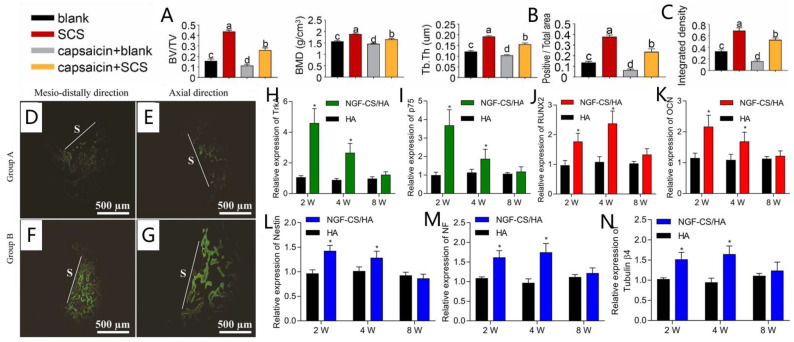
Surface alteration influences osseointegration and innervation. (**A**–**C**): Through sensory nerve innervation, SCS stimulates bone repair [205]. (**A**) The SCS group had substantially greater BV/TV, Tb, Th, and BMD values than the other three groups; (**B**) Von Kossa staining; and (**C**) in vivo calcein labeling. (**D**–**G**): Using the CTAN software tool, 3D pictures of beagle maxilla samples were created to assess bone development in tooth defects [206]. The fluorochrome labeling of calcein (green) injected on week 2 consistently demonstrated that group B animals (**F**,**G**) had more bone regeneration than group A animals (**D**,**E**). (**H**–**N**): NGF, osteogenesis differentiation, and neurogenic differentiation-related genes were significantly elevated when NGF-CS/HA-coating implants were implanted [207]. RT-qPCR analysis of Trk A and p75 (**H**,**I**); OCN and Runx-2 (**J**,**K**); and Nestin, NF, and tubulin β4 (**L**–**N**) in HA- and NGF-CS/HA-coating implant groups at 2, 4, and 8 weeks. * *p* < 0.05. Different lowercase letters indicate significant differences.

The regenerative potential of the nervous system for osseointegration has been noticed, and some researchers continue to modify the material’s surface to affect the nervous system and improve osseointegration. The de novo bone formation was abundant when silicified collagen scaffolds were implanted into wounds created in rats’ far-end femurs. There was significant nerve innervation and angiogenesis at the wound sites. Silicon appears to have therapeutic promise in orthopedic rehabilitation, according to this study (Figure 4A–C) [205]. The researchers employed multi-electrode stimulation of the sympathetic nervous system in the infraorbital nerve after implanting cylindrical titanium implants inside the tooth sockets of beagles for one week. Microelectrode stimulation of the sympathetic nervous system, a simple and safe physical method, may aid in bone formation and the osseointegration of implants, according to the findings (Figure 4D–G) [206]. Another study generated NGF-CS/HA-coating composite implants, which substantially elevated NGF, osteogenesis differentiation, and neurogenic differentiation-related genes in the mandibles of Beagle dogs after implantation (Figure 4H–N) [207]. In rats with ovariectomy-induced osteoporosis, researchers produced a novel magnesium-containing intramedullary nail that boosted CGRP-mediated osteogenic differentiation and facilitated femur fracture healing [208]. The fracture gaps were filled using pasty hydroxyapatite-forming bone cement integrating mesoporous bioactive CaP-SiO_2_ glass particles with and without BDNF, and the degree of regeneration was measured after 5 weeks. Compared to WT mice that did not receive the BDNF-functionalized cement/MBG composite, the bone formation in the WT mice who received the BDNF-functionalized cement/MBG composite was considerably increased [209].

Above all, while some researchers have yielded promising outcomes, the mechanism of surface modification on osseointegration and innervation remains a mystery. The surface modification of materials should be pursued further for the advanced study of the nervous system, and its mechanism should be unraveled.

## 6. Conclusions

A vast amount of research and evidence has demonstrated that neurotrophins, neuropeptides, and nerve cells participate in bone formation, bone regeneration, and other bone-related activities. The nervous system has an impact on osseointegration at various stages. For example, the inflammatory response is mediated by several neurotrophins and neuropeptides. They have a variety of effects, including increasing immune cell proliferation and influencing antibody synthesis, among others. Some neurotrophins and neuropeptides work together to promote neovascularization and angiogenesis. After implantation, they can also facilitate osseous development and remodeling during osseointegration. Accordingly, few researchers have launched pioneering studies into the surface-modification of the implant material to manipulate the nervous system and promote osseointegration. As a result, this review underlines the relevance of the nervous system in osseointegration and calls for a possible approach to improve osseointegration from a novel viewpoint.

## Figures and Tables

**Table 1:** The receptors and effect of neurotrophins on bone.

Neurotrophin	Receptor	Effect	Ref.
NGF	TrkA, p75	Mediating the bone’s response to mechanical loading; enhances bone regeneration via regulation of osteogenesis and bone resorption	[58,59,60,61,62,63]
NT-3	TrkC	Improves bone-fracture healing by improving the formulation of osteoblasts and augments osteoclastogenesis and resorption; promote heterotopic ossification formation	[64,65,66,67]
NT-4/5	TrkB	Regulates the functions of periodontal ligament cells	[68,69,70,71,72]
BDNF	TrkB	Regulates new bone formation by inducing osteoblast proliferation and activating osteoclasts	[59,73,74,75,76,77]
GDNF	RET, GFRα-1, and GFRα-2	Involved in the pathogenesis of bone pain; regulates bone metabolism; acts as a target-derived neurotrophic factor during tooth innervation	[78,79,80,81,82]
PDGF	type A PDGF receptor, type B PDGF receptor	Improves reparative osseous activity; promote angiogenesis	[83,84,85,86]
FGF	FGFR	Improves fracture healing; stimulates bone resorption; promote Angiogenesis	[87,88]

**Table 2:** The effect of neuropeptides on bone.

Neuropeptides	Effect	Ref.
CGRP	Transmits pain and sensitization; is involved in fracture healing; promotes bone growth and inhibits bone resorption	[101,102,103,104,105,106,107,108,109,110,111,112,113]
SP	Collaborates in callus development by increasing local blood flow; affects the metabolism of bones by directly impacting bone cells and affects blood vessels and the generation of other cytokines	[114,115,116,117,118]
VIP	Aids bone fracture repair by stimulating osteoblastic activity and decreasing osteoclastic activity; improves bone density and mechanical features	[119,120,121,122]
PACAP	Has anti-inflammatory and chondroprotective properties	[123]
NPY	Controls bone resorption by increasing OPG expression in osteoblasts and inhibiting isoprenaline-induced osteoclastogenesis	[124,125,126]

## Data Availability

The data will be available from the corresponding author following reasonable request.
